# Deep EST profiling of developing fenugreek endosperm to investigate galactomannan biosynthesis and its regulation

**DOI:** 10.1007/s11103-012-9909-y

**Published:** 2012-04-17

**Authors:** Yan Wang, Ana P. Alonso, Curtis G. Wilkerson, Kenneth Keegstra

**Affiliations:** 1 DOE Great Lakes Bioenergy Research Center, Michigan State University, East Lansing, MI 48824 USA; 2 MSU-DOE Plant Research Laboratory, Michigan State University, East Lansing, MI 48824 USA; 3 Department of Plant Biology, Michigan State University, East Lansing, MI 48824 USA; 4 Department of Biochemistry and Molecular Biology, Michigan State University, East Lansing, MI 48824 USA; 5 Present Address: Department of Molecular Genetics, Ohio State University, Columbus, OH 43210 USA

**Keywords:** Fenugreek, Endosperm, Galactomannan biosynthesis, EST profiling, Hexose phosphates, Nucleotide sugars

## Abstract

Galactomannans are hemicellulosic polysaccharides composed of a (1 → 4)-linked *β*-D-mannan backbone substituted with single-unit (1 → 6)-α-linked D-galactosyl residues. Developing fenugreek (*Trigonella foenum*-*graecum*) seeds are known to accumulate large quantities of galactomannans in the endosperm, and were thus used here as a model system to better understand galactomannan biosynthesis and its regulation. We first verified the specific deposition of galactomannans in developing endosperms and determined that active accumulation occurred from 25 to 38 days post anthesis (DPA) under our growth conditions. We then examined the expression levels during seed development of *ManS* and *GMGT*, two genes encoding backbone and side chain synthetic enzymes. Based on transcript accumulation dynamics for *ManS* and *GMGT*, cDNA libraries were constructed using RNA isolated from endosperms at four ages corresponding to before, at the beginning of, and during active galactomannan deposition. DNA from these libraries was sequenced using the 454 sequencing technology to yield a total of 1.5 million expressed sequence tags (ESTs). Through analysis of the EST profiling data, we identified genes known to be involved in galactomannan biosynthesis, as well as new genes that may be involved in this process, and proposed a model for the flow of carbon from sucrose to galactomannans. Measurement of in vitro ManS and GMGT activities and analysis of sugar phosphate and nucleotide sugar levels in the endosperms of developing fenugreek seeds provided data consistent with this model. In vitro enzymatic assays also revealed that the ManS enzyme from fenugreek endosperm preferentially used GDP-mannose as the substrate for the backbone synthesis.

**Electronic supplementary material:**

The online version of this article (doi:10.1007/s11103-012-9909-y) contains supplementary material, which is available to authorized users.

## Introduction

Plant cell walls constitute the most abundant biomass on Earth, mainly composed of polysaccharides. Plant wall polysaccharides consist of cellulose and matrix polysaccharides, including hemicellulose and pectin. Cellulose is the most abundant polysaccharide, normally constituting 30–50 % of the wall dry mass (Pauly and Keegstra 2008). Hemicellulose is the second most abundant component of plant walls, making up 20–35 % of the wall material (Pauly and Keegstra 2008). Based on compositional and structural differences, hemicellulose is classified into xyloglucans, xylans, mannans and mixed-linkage (1 → 3),(1 → 4)-β-d-glucans (Scheller and Ulvskov 2010).

Mannan polysaccharides are present in all land plants studied so far. Several types of mannan polymers have been found: mannans, glucomannans, galactomannans and galactoglucomannans (Scheller and Ulvskov 2010). Mannans contain a (1 → 4)-β-linked backbone composed of mannose (Man), whereas glucomannans contain a backbone composed of both Man and glucose (Glc). Substitutions of mannosyl residues of the mannan or glucomannan backbone by single-unit (1 → 6)-α-linked galactose (Gal) give rise to galactomannans or galactoglucomannans (Scheller and Ulvskov 2010). Mannan polysaccharides are not evenly distributed in different tissues of plants, and their content varies greatly among plants. Glucomannans are the main hemicellulose in the secondary walls of gymnosperms (Fengel and Wegener 1989). Galactomannans accumulate in large quantities in the seed endosperms of many leguminous plants such as guar (*Cyamopsis tetragonoloba*) and fenugreek (Reid 1985).

The degree of substitution of the mannan backbone by Gal or the Man/Gal ratio determines the water solubility of mannan polysaccharides. Pure mannans are insoluble in water, and mannan polymers with a high degree of galactosylation (low Man/Gal ratio) have high solubility (Reid 1985). Galactomannan gums from seeds of different plants differ by Man/Gal ratios (Reid 1985), and thus exhibit different chemical properties, including water holding, thickening, gelling, binding, suspending, emulsifying, and formation of films (Srivastava and Kapoor 2005). These characteristics make galactomannans widely used as versatile materials in industries such as food, textiles, paper, pharmaceutics, cosmetics, petroleum, drilling, or explosives (Srivastava and Kapoor 2005). Moreover, mannan polysaccharides may be a good target for improving plant feedstock for biofuel production. Galactomannans are water soluble, and thus more accessible to enzymatic degradation, compared with cellulose microfibrils. The released sugars, Man and Gal, are both hexoses, which are easily fermentable compared with pentoses. It is therefore attractive to consider increasing the galactomannan contents in vegetative tissues of bioenergy crop plants to enhance biofuel production (Pauly and Keegstra 2008).

Like other hemicellulosic polysaccharides, mannans are synthesized in the Golgi, in contrast to cellulose, which is synthesized at the plasma membrane. The backbone of mannan polysaccharides is synthesized by mannan synthase (ManS) (Dhugga et al. 2004; Liepman et al. 2005), and addition of single-unit galactosyl residues to Man is catalyzed by galactomannan galactosyltransferase (GMGT) (Edwards et al. 1999). The *ManS* gene was first identified from guar as a member of the *cellulose synthase*-*like A* (*CSLA*) family of genes (Dhugga et al. 2004). *CSLA* genes have been found in all land plants (Yin et al. 2009). When expressed in a heterologous system, CSLA proteins have been shown to synthesize the mannan or glucomannan backbone in vitro (Dhugga et al. 2004; Gille et al. 2011; Liepman et al. 2007; Liepman et al. 2005; Suzuki et al. 2006). Analysis of *CSLA* mutants in *Arabidopsis* provided further evidence that CSLA proteins are responsible for glucomannan biosynthesis in vivo (Goubet et al. 2009). *GMGT* was first identified from fenugreek and has been functionally characterized (Edwards et al. 1999, 2002, 2004; Reid et al. 2003).

To better understand the galactomannan biosynthetic pathway and its regulatory mechanism, we constructed cDNA libraries using RNA isolated from the endosperms of developing fenugreek seeds at four ages spanning the period of active *ManS* and *GMGT* transcript accumulations and corresponding to before, at the beginning of, and during active galactomannan deposition. By deep sequencing of the cDNA libraries, we identified genes postulated to act in the galactomannan biosynthetic pathway and proposed a model for how it operates. We also performed in vitro enzymatic activity assays of fenugreek endosperm ManS and GMGT to confirm that these activities increased consistent with transcript levels and galactomannan deposition during endosperm development. In addition, in vitro enzymatic activity assays using isolated fenugreek endosperm microsomes revealed that only GDP-Man could be efficiently used as the substrate for the backbone synthesis. Finally, we analyzed the sugar phosphate and nucleotide sugar levels in the endosperms of developing fenugreek seeds to evaluate our model. In addition to genes proposed to act in the galactomannan biosynthetic pathway, we also identified genes for sugar transporters, transcription factors and an unknown protein containing Domain of Unknown Function 246 (DUF246), which may potentially be involved in mediating or regulating galactomannan biosynthesis.

## Materials and methods

### Plant growth and tissue collection

Fenugreek plants were grown essentially as described in Alonso et al. (2010), using an approach modified based on Edwards et al. (1989). Flowers were tagged at anthesis. Developing seeds or dissected seed tissues were harvested at appropriate ages as experimental materials.

### Preparation of AIR (alcohol insoluble residue) samples

For analysis of fenugreek single developing seeds, the second seed from the proximal end of each pod from three individual plants was harvested at each age. For analysis of different seed tissues, single endosperms, embryos and seed coats were dissected from the second seed from the proximal end of each pod at different ages. The harvested seeds or tissues were immediately frozen in liquid nitrogen and stored at −80 °C before being used for preparation of AIR samples.

AIR samples were prepared by a method from Cavalier et al. (2008) with some modifications. Briefly, 1 mL 70 % (v/v) ethanol was added to each tube containing a frozen single seed or a frozen single seed tissue, and the tube was then incubated at 65 °C for at least 1 h to inactivate enzymes. The single seed or tissue was ground in a glass homogenizer. The homogenized sample was centrifuged, and the pellet was washed twice with 70 % (v/v) ethanol. The pellet was resuspended in 0.5 mL 70 % ethanol, transferred to a preweighed 2-mL tube with screw cap, and vacuum dried. The AIR was weighed and suspended in 1 mL water.

### Neutral monosaccharide composition analysis

A portion of the AIR suspension was transferred into a glass vial, and 5 μg of inositol was then added as an internal standard. The sample was vacuum dried. The dried sample was subjected to trifluoroacetic acid hydrolysis, reduction to alditols, acetylation, extraction and gas chromatography/mass spectrometry analysis, following the protocol of Cavalier et al. (2008).

### RNA isolation and Northern blot analysis

Fenugreek tissues (seeds, endosperms, embryos and leaves) were harvested, frozen in liquid nitrogen and stored at −80 °C until use. The frozen tissues were ground into fine powders in liquid nitrogen with a mortar and pestle. RNA was isolated from the ground tissues, according to Lopez-Gomez and Gomez-Lim (1992), with some modifications described below. Following homogenization with a PowerGen 700 polytron (Fisher Scientific), the tissue homogenate was incubated at 65 °C for 10 min before adding ethanol and potassium acetate. After precipitation of RNA with LiCl and centrifugation, the RNA pellet was washed with 3 M LiCl once and then with 70 % (v/v) ethanol once. The RNA pellet was briefly air-dried and dissolved in ribonuclease-free water. The RNA was treated with DNase I and purified using the RNeasy Plant Mini Kit (Qiagen).

Northern blot analysis was conducted prior to sequencing ESTs from fenugreek endosperms. Because fenugreek *ManS* had not previously been sequenced, we cloned its cDNA by RT-PCR using RNA isolated from fenugreek endosperms. We designed multiple pairs of primers based on the conserved regions of *ManS* cDNA and EST sequences from guar (Dhugga et al. 2004) and other species available at GenBank (http://www.ncbi.nlm.nih.gov/Genbank/), and tested them in RT-PCR. One pair of primers (5′-GAGGATATGGACCTTGCAGT-3′ and 5′-GCACAGTGCAGCATATACAT-3′) successfully amplified a 0.6-kb *ManS* cDNA fragment. The cloned *ManS* cDNA fragment and the fenugreek *GMGT* cDNA (Edwards et al. 1999) were used as probes in Northern blot analysis following the protocol of Sijacic et al. (2004). Approximately 10 μg of total RNA isolated from fenugreek whole seeds, leaves or 32 DPA embryos and 2 μg of total RNA from 32 DPA endosperms were loaded.

To quantify Northern blot data, images on x-ray films were scanned, and the intensity of hybridization signals and rRNA bands (from the ethidium bromide-stained RNA gel) was measured using the Quantity One version 4.6.5 software (Bio-Rad). The relative transcript level for each age was calculated as a ratio of the intensity of a hybridization band to the intensity of 18 and 28 s rRNA bands. The 25-DPA age was used as a reference age.

### Real-time quantitative RT-PCR

Total RNA was isolated from fenugreek endosperms of different ages as described above. First-strand cDNA was synthesized from the total RNA using SuperScript III Reverse Transcriptase (Invitrogen).

Fenugreek *EF*-*1*-*α* cDNA was cloned by RT-PCR from endosperm RNA using the same strategy as described in the cloning of *ManS* cDNA. After testing multiple primer pairs, one pair of primers (5′-ATGGGAAAAGAAAAGATTCATAT-3′ and 5′-GTCTC(A/C)ACAACCATGGGCTTGGT-3′) were chosen to amplify a 1.1-kb *EF*-*1*-*α* cDNA fragment. The cloned *EF*-*1*-*α* and aforementioned *ManS* cDNA fragments were sequenced. Based on the cDNA sequences of fenugreek *ManS*, *GMGT* (Edwards et al. 1999) and *EF*-*1*-*α*, the corresponding primers were designed using Primer Express version 1.0 (Applied Biosystems).

The relative expression of *ManS* and *GMGT* was examined by real-time quantitative RT-PCR using the SYBR Green PCR Master Mix (Applied Biosystems) with the fenugreek endosperm first-strand cDNA as a template. Primers used for PCR analysis were: 5′-GACGCGGCTTCAAGAGATGT-3′ and 5′-TTGCTTGAATCCGCCAAATT-3′ for *ManS*, 5′-GGTCGACTCTGATGCCATCTTT-3′ and 5′-ACCAACTCTTCCCAACCATGAA-3′ for *GMGT*, and 5′-TCCTCCATTGGGACGTTTTG-3′ and 5′-CTTGGCTCCGGTAGGATCCT-3′ for *EF*-*1*-*α*. PCR was performed with an ABI PRISM 7900 HT Sequence Detection System (Applied Biosystems). Data were analyzed with the SDS 2.3 software (Applied Biosystems). The PCR threshold cycle number of *ManS* or *GMGT* was normalized with that of the *EF*-*1*-*α* reference gene to calculate the relative mRNA levels. The 20-DPA age was used as a reference age.

### Endosperm cDNA library construction and 454 FLX sequencing

Based on the quantitative RT-PCR data, fenugreek cDNA libraries were constructed from endosperm RNA of 20, 25, 28 and 32 DPA with the SMART cDNA Library Construction Kit (Clontech), following the manufacturer’s instructions except that a modified CDS III/3′ PCR Primer (TAGAGGCCGAGGCGGCCGACATGTTTTGTTTTTTTTTCTTTTTTTTTTVN) was used. The cDNA libraries were sequenced by the Joint Genome Institute of the US Department of Energy (www.jgi.goe.gov/), using a Roche GS-FLX sequencer (454 Life Sciences).

### Assembly of EST reads and annotation of consensus sequences

EST data from all four cDNA libraries were used to assemble contigs essentially as described by Durrett et al. (2010). The consensus sequences were compared to the *Arabidopsis* (http://www.arabidopsis.org/) and Swiss-Prot (http://ca.expasy.org/sprot/) protein databases using the program BLASTX. BLASTX results were stored in an Oracle relational database to facilitate assignment of probable gene function.

The database for consensus sequences of fenugreek EST contigs and their annotation is available at the website http://glbrc.bch.msu.edu/fenugreek. To find genes of interest, the EST database was searched using a key word (e.g. a gene name) or an *Arabidopsis* gene identity (AGI) number. The database was also searched with the BLASTN or TBLASTN program using a nucleotide or protein sequence as a query to find homologous sequences.

In some cases where ESTs from one gene were assembled into at least 2 different contigs, the sequence of one contig was used as a query to search the EST database to find the other overlapping contig(s). The complete consensus cDNA sequence of the gene was deduced from the overlapping contig sequences, and the EST read number and expression level of the gene were calculated by totaling that of the overlapping contigs.

After elimination of contigs for rRNA sequences, we recalculated the expression level of each endosperm-expressed gene, and normalized it as parts per million (ppm).

### Preparation of fenugreek endosperm homogenate and enzymatic activity assays of ManS and GMGT

Fenugreek endosperm homogenate preparation and ManS and GMGT assays were carried out as described by Edwards et al. (1989) with modifications. Briefly, endosperms were dissected out from seeds, and immediately dropped into ice-cold Isolation Buffer (50 mM Tris–HCl, pH 7.5, 5 mM MgCl_2_, 5 mM DTT) supplemented with protease inhibitors (one Complete-Mini EDTA-Free Protease Inhibitor Tablet per 10 mL buffer; Roche). The endosperms were homogenized in a glass homogenizer at 4 °C, and fresh homogenate was directly used for ManS and GMGT enzymatic assays. For both assays, the 100 μL reaction mixture comprised 50 μL homogenate, 20 μL 5 × Assay Buffer (125 mM Tris–HCl, pH 7.5, 12.5 mM MgCl_2_, 25 mM MnCl_2_, and 12.5 mM DTT), 80 μM GDP-Man, and 800 μM UDP-Gal. For ManS assay, the 80 μM GDP-Man included 1.53 μM GDP-[^14^C]Man (PerkinElmer) at 185 Bq nmol^−1^, and for GMGT assay, the 800 μM UDP-Gal included 5 μM UDP-[^14^C]Gal (Amersham Biosciences) at 71 Bq nmol^−1^. The reaction mixture was incubated at room temperature for 1 h, except for the time course assays. Termination of the reactions, precipitation of reaction products, and washing of pellets were carried out as described by Liepman et al. (2005). The washed pellets were resuspended in 300 μL H_2_O. The suspension was transferred to a 4-ml plastic scintillation vial, and 3 mL Optiphase Supermix scintillation fluid (PerkinElmer) was then added to the vial. After mixing, the sample was subjected to liquid scintillation counting using the1450 MicroBeta Trilux liquid scintillation counter (PerkinElmer).

### Protein expression in *Pichia pastoris* cells and isolation of microsomes

Based on the sequence of the fenugreek *ManS* (*TfManS*) cDNA contig assembled from EST reads generated by 454 sequencing, two primers were designed, 5′-CACCATAATGGCTAGCATGACTGGTGGACAGCAAATGGGTATGAGAAACCTAATATTTGAGGAGCC-3′ (containing a Kozak sequence “CATA” and a T7 tag sequence “ATGGCTAGCATGACTGGTGGACAGCAAATGGGT” upstream of the gene-specific region) and 5′-TTAGTTGGGTACAATTGTTCCCAC-3′. The primers were used to amplify the N-terminus T7-tagged coding region of *TfManS* from fenugreek endosperm RNA by RT-PCR. The T7-tagged coding region was subcloned into the pPICZ vector for *Pichia* expression as described by Davis et al. (2010). Transformation of the *TfManS* expression construct into *Pichia* cells, identification of transformants containing multiple copies of the transgene, and expression of proteins in *Pichia* cells were performed according to Davis et al. (2010). The *Pichia* strains expressing T7-tagged AtCSLA9 and AtFUT1 (*Arabidopsis* xyloglucan fucosyltransferase) proteins (Davis et al. 2010) were kindly provided by Jonathan Davis.

For isolation of microsomes, cells were pelleted from 30 mL of saturated *Pichia* cultures and washed twice with ice-cold water as described by Davis et al. (2010). Cells were broken using a glass bead approach according to the instruction manual of EasySelect Pichia Expression Kit (Invitrogen) with modifications. Pelleted cells were resuspended in 4 mL of the extraction buffer (50 mM HEPES–KOH, pH 7.5) supplemented with protease inhibitors (one Complete-Mini EDTA-Free Protease Inhibitor Tablet per 10 mL buffer; Roche). After addition of 2 mL of acid-washed glass beads (Sigma), cells were vortexed vigorously for 30 s for a total of 8 cycles, incubating samples on ice for 30 s between cycles. The cell lysate was centrifuged at 12,000×*g* for 15 min at 4 °C, and the resulting supernatant was centrifuged at 120,000×*g* for 1 h at 4 °C to pellet microsomal membranes. Membranes were resuspended in 600 μL of the extraction buffer. Microsomal samples were frozen in liqud nitrogen and stored at −80 °C until use.

### Enzymatic assays of ManS, GlcS (glucan synthase) and GlcManS (glucomannan synthase)

To test the substrate specificity for the backbone synthesis of fenugreek endosperms, microsomal membranes were isolated from the homogenate of 35-DPA endosperms prepared as described above. The homogenate was centrifuged at 1,000×*g* for 10 min, and the resulting supernatant was then centrifuged at 120,000×*g* for 1 h. The microsomal membrane pellet was resuspended in Isolation Buffer (250 μL per endosperm). Microsomes were stored at −80 °C until use. The assays were conducted at room temperature for 1 h according to Liepman et al. (2005). The 40 μL reaction mixture contained 20 μL microsomal membranes, 10 μL 4 × assay buffer (100 mM Tris–HCl, pH 7.5, 10 mM MgCl_2_, 20 mM MnCl_2_, and 10 mM DTT), and cold and radio-labeled GDP-Man and/or GDP-Glc. The concentrations of substrates for the various assays were as follows (concentration of radiolabeled substrate in parentheses): ManS, 25 μM GDP-Man (1.9 μM); Glc*ManS, 25 μM GDP-Man, 25 μM GDP-Glc (2.1 μM); GlcMan*S, 25 μM GDP-Man (1.9 μM), 25 μM GDP-Glc; GlcS, 25 μM GDP-Glc (2.1 μM). Reactions were terminated, and the products were pelleted, washed and subjected to liquid scintillation counting as described above.

To test the substrate specificity for the backbone synthesis of heterologously expressed TfManS and AtCSLA9, *Pichia* microsomes expressing the two proteins and AtFUT1 (used as a control) were used in the assays. The assays were conducted as described above. The 40 μL reaction mixture contained 10 μL microsomal membranes, 1 × assay buffer, and cold and radio-labeled GDP-Man and/or GDP-Glc. The concentrations of substrates for the assays were as follows: ManS, 25 μM GDP-Man (3.8 μM); Glc*ManS, 25 μM GDP-Man, 25 μM GDP-Glc (4.0 μM); GlcMan*S, 25 μM GDP-Man (3.8 μM), 25 μM GDP-Glc; GlcS, 25 μM GDP-Glc (4.0 μM). Assay products were analyzed as described above.

### Analysis of sugar phosphates and nucleotide sugars

To avoid carbonate contamination, NaOH was purchased from Fluka in liquid form. All the water used as eluent and reagent was deionized and degassed. The LC was performed with an ACQUITY UPLC^®^ pump system (Waters). The eluents were vacuum degassed. Samples in the autosampler were kept at 4 °C, whereas the LC analysis was carried out at room temperature. Phosphorylated metabolites were separated by ion chromatography on an IonPac^®^ AS11 (250 mm × 2 mm, Dionex) column equipped with a guard column AG11 (50 mm × 2 mm, Dionex) at a flow rate of 0.35 mL min^−1^, as previously described by Alonso et al. (2010).

The MS/MS analyses were performed with a Quattro-Premier^™^ (Waters), triple quadrupole mass spectrometer. Mass spectra were acquired using electrospray ionization in negative ion mode and multiple reaction monitoring. The capillary voltage, extractor voltage, and rf lens setting were set at 3.00 kV, 5 V, and 0.0, respectively. The flow rates of cone gas and desolvation gas were 50 and 800 L h^−1^, respectively. The source temperature and desolvation temperature were 100 and 350 °C, respectively. The [M–H]^−1^ were fragmented by collision-induced dissociation with argon as collision gas at a manifold pressure of 2.67 × 10^−3^ mbar. Collision energies and source cone potentials were optimized for each transition using Waters QuanOptimize software (Alonso et al. 2010). Data were acquired with MassLynx 4.0 and processed for calibration and for quantification of the analytes with QuanLynx software.

Intracellular metabolites were extracted from fenugreek endosperms of 20, 25, 28 and 32 DPA using boiling water as previously described by Alonso et al. (2010). Dried extracts were resuspended in 100 μL of Milli-Q water to be analyzed by LC–MS/MS.

## Results

### Neutral monosaccharide composition analysis of developing fenugreek seeds and different seed tissues

**Fig. 1:**
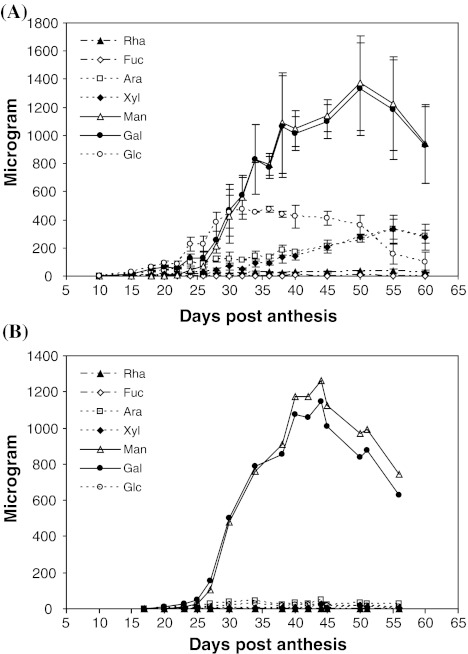
Neutral monosaccharide composition analysis. **a** Monosaccharide composition of single seeds. Data for each age are from three biological replicates, with one measurement of a single seed (the second seed from the proximal end of each pod) for each replicate. *Error bars* show the standard deviation at each age. **b** Monosaccharide composition of single endosperms. The analysis was performed for single endosperms (from the second seed from the proximal end of each pod) from four individual plants; representative data from only one plant are shown for clarity, with one measurement for one single endosperm at each age. Ara, arabinose; Fuc, fucose; Gal, galactose; Glc, glucose; Man, mannose; Rha, rhamnose; Xyl, xylose

We sought to determine the timing of galactomannan deposition in developing fenugreek seeds under our growth conditions in comparison with that reported by Edwards et al. (1992). We first prepared AIR from individual developing seeds and the AIR samples were subjected to neutral monosaccharide composition analysis (Fig. 1a). We analyzed individual seeds in an attempt to understand the seed to seed variation in the timing of galactomannan deposition. Gal and Man were the two predominant monosaccharides in developing seeds. There was also a significant amount of Glc at certain stages of development (Fig. 1a) probably derived from starch. The Gal and Man contents slowly increased from 15 to 26 DPA, but increased dramatically from 26 to 38 DPA, reaching their maximum levels at 50 DPA, and decreasing slightly at later times. These results are consistent with previous reports (Edwards et al. 1992), but under our growth conditions, active galactomannan deposition occurred earlier, beginning at 26 DPA instead of 32 DPA, as reported by Edwards et al. (1992). The combined mass of Gal and Man at later stages was about 0.3 mg per mg of the AIR (Fig. 1a; data not shown), consistent with the previous report that galactomannans constitute 30 % of the dry weight of the seed (Reid and Bewley 1979).

To verify the specific deposition of galactomannans in the endosperm during seed development, we performed neutral monosaccharide composition analysis of endosperms from four plants. Active galactomannan deposition occurred from 25 to 40 DPA (Fig. 1b), consistent with the data from whole seeds (Fig. 1a). Man and Gal together constituted up to 98 % of the total non-cellulose neutral monosaccharides in the endosperm at late ages (Fig. 1b). The molar ratio of Man to Gal increased during development and reached a plateau at 40 DPA, with the maximum value of 1.1 (Fig. 1b; data not shown), which is consistent with an earlier report (Reid and Meier 1970b). For one of the plants, single embryos and seed coats were also analyzed, and the amounts of Man and Gal were negligible (data not shown). Our data provide further biochemical evidence to confirm the specific deposition of galactomannans in the endosperm, as previously demonstrated by microscopy studies of fenugreek seed (Meier and Reid 1977; Reid 1971), and are consistent with the conclusions obtained with guar seed (Dhugga et al. 2004).

### Examination of the transcript levels of *ManS* and *GMGT* genes

Two enzymes catalyze galactomannan biosynthesis; ManS forms the β-1,4-linked mannan backbone using GDP-Man as the donor substrate, and GMGT adds the α-1,6-galactosyl side chains using UDP-Gal as the donor substrate. A fenugreek gene encoding the GMGT enzyme was identified and cloned by Edwards et al. (1999). *ManS* genes have been identified and cloned from other species (Dhugga et al. 2004; Liepman et al. 2005, 2007; Pre et al. 2008; Suzuki et al. 2006). To further study galactomannan biosynthesis at the molecular level, we examined the transcript levels of these two genes during seed development by Northern blot analysis, using RNA isolated from fenugreek developing seeds. The *GMGT* transcript was first detected at 25 DPA, peaked at 30 DPA, and became undetectable after 50 DPA. The *ManS* transcript was barely detectable at 25 DPA, peaked at 35 DPA, and decreased afterwards (Supplemental Fig. 1).

**Fig. 2:**
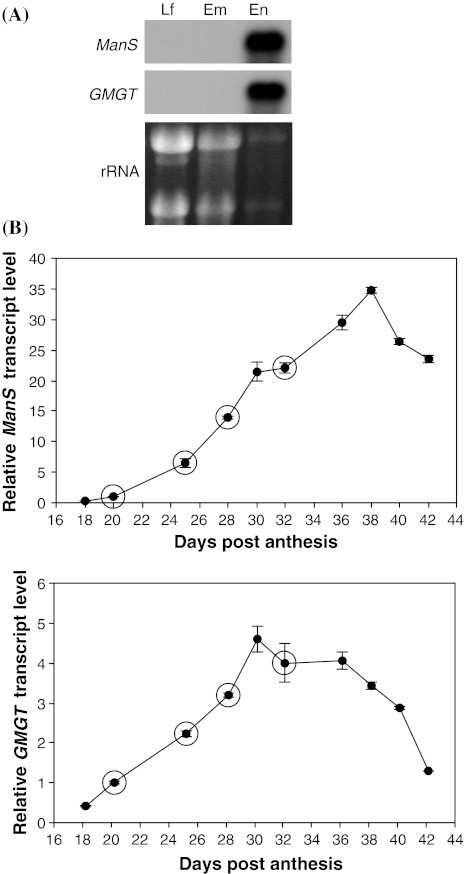
Expression of *ManS* and *GMGT* genes. **a** Northern blot analysis of *ManS* and *GMGT* using total RNA isolated from fenugreek leaf (Lf), embryo (Em) and endosperm (En) tissues. The rRNA bands from the ethidium bromide stained agarose gel are shown as loading controls. **b** Quantitative RT-PCR analysis showing relative *ManS* and *GMGT* transcript levels in the endosperm of developing seeds. Ratios are expressed relative to the 20 DPA (days post anthesis) age. *Error bars* represent the standard deviation of three replicate measurements for the same RNA sample. *Circles* indicate the four ages at which RNA samples were used for cDNA library construction and 454 sequencing

To investigate tissue-specific expression of *ManS* and *GMGT*, Northern blot analysis was conducted using RNA isolated from three fenugreek tissues, leaves, embryos and endosperms. Both genes were highly expressed only in the endosperm (Fig. 2a), consistent with the endosperm-specific expression of *ManS* in guar, another leguminous species, which also accumulates galactomannans as storage carbohydrates in seed endosperm (Dhugga et al. 2004).

To further quantify the expression levels of *ManS* and *GMGT* during seed development, quantitative RT-PCR analysis was conducted using RNA isolated from endosperms at different ages. The transcript levels of both genes increased from as early as 18 DPA, but active transcript accumulation for *ManS* (from 20 to 38 DPA) started later than that for *GMGT* (from 18 to 30 DPA). *ManS* expression peaked at 38 DPA, whereas *GMGT* expression peaked at 30 DPA, before declining (Fig. 2b). These data are consistent with the Northern blot data generated from whole seeds (Supplemental Fig. 1).

### EST profiling of fenugreek seed endosperm

Based on the quantitative RT-PCR results, endosperm RNA at 20, 25, 28 and 32 DPA was used to construct cDNA libraries. The four ages corresponded to the times before, at the beginning of, and during active galactomannan deposition (Fig. 1). The four cDNA libraries were sequenced with a Roche GS-FLX sequencer (454 Life Sciences). As a result, after excluding reads for rRNA, a total of 1.5 million ESTs were generated with an average size of 216 bp, at least 0.3 million ESTs for each age. The sequence information (accession numbers SRX026988–SRX026992) was deposited in the sequence read archive at the National Center for Biotechnology Information (http://www.ncbi.nlm.nih.gov/).

The EST reads were assembled into contigs, and the consensus sequences were annotated by homology to genes from *Arabidopsis* and other species using the TAIR8 and Swiss-Prot protein databases and the program BLASTX. These data were stored in an Oracle relational database and can be viewed using a web-based viewer (http://glbrc.bch.msu.edu/fenugreek/). After eliminating the reads for rRNA sequences, the expression levels of endosperm-expressed genes were recalculated and shown as ppm. Supplemental Table 1 lists contigs with at least 50 EST reads. Through analysis of the contig sequences, we identified approximately 1,650 fenugreek endosperm-expressed genes with at least 100 EST reads. Hierarchical cluster analysis (Eisen et al. 1998) was then used to analyze the expression patterns of these genes during seed development (20–32 DPA). Approximately 40 % of them were up-regulated during endosperm development (Supplemental Fig. 2a).

Table 1 summarizes the 10 most highly expressed fenugreek transcripts, ranked by their numbers of EST reads. Among the top 10 transcripts were those encoding ManS and GMGT, providing support for the hypothesis that transcriptional profiling of this tissue should reveal genes necessary for galactomannan biosynthesis. The highly expressed genes also included those encoding a lipid transfer protein, a seed storage protein, protease inhibitors, a cysteine-rich protein, a peroxidase, a hypothetical protein and an unknown protein containing DUF246. The transcript levels for all 10 genes increased by greater than four-fold during development (from 20 to 28 DPA).

**Table 1:** Top 10 fenugreek endosperm-expressed genes ranked by total number of EST reads

Rank	ESTs	Fenugreek gene	*Arabidopsis*	Expression (ppm)			
			Best hit	20 DPA	25 DPA	28 DPA	32 DPA
1	50478^b^	Non-specific lipid-transfer protein	*At4g33355*	1,685	34,015	53,311	42,695
2	33061^b^	Unknown protein (*DUF246*)	*At3g21190*	5,042	23,644	28,966	31,055
3^a^	26236	Hypothetical protein		454	18,475	29,528	18,845
4	17620	Cupin family protein (7S SSP family)	*At3g22640*	438	9,913	20,016	15,080
5	16815	Cationic peroxidase	*At4g21960*	3,710	13,419	15,486	11,506
6	14803	LMW cysteine-rich protein (peptidase inhibitor)	*At2g02120*	25	3,951	14,961	21,187
7	14239^b^	Galactomannan galactosyl transferase (*GMGT*)	*At2g22900*	2,761	10,785	11,641	13,285
8	11836	SCR (*S* locus cysteine-rich protein)-like protein	*At4g15735*	2,149	12,613	11,559	3,944
9	11739	Cystatin (cysteine protease inhibitor)	*At5g47550*	262	8,704	13,798	6,901
10	6230^b^	Mannan synthase (*ManS*)	*At5g03760*	500	2,885	5,031	8,934

*DPA* days post anthesis, *LMW* low-molecular-weight, *SSP* seed storage protein

^a^Most similar to an uncharacterized protein (MTR_3g034220) of *Medicago truncatula* in the non-redundant protein database, but no significant *Arabidopsis* hit found

^b^Total number of EST reads added from that of two different contigs corresponding to the same gene

**Fig. 3:**
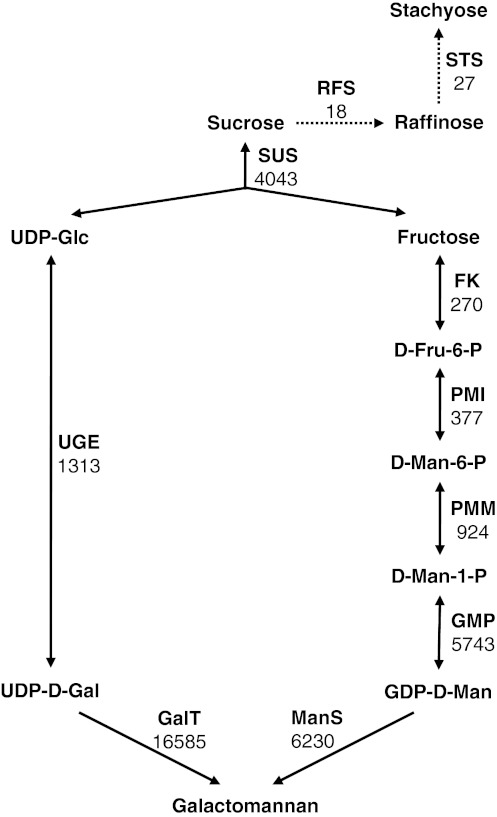
Model for galactomannan biosynthetic pathway. The steps for biosyntheses of raffinose and stachyose are also included for comparison and shown in *dash lines* with arrowheads. The number under each enzyme represents the total number of EST reads for the corresponding gene (family). *RFS* raffinose synthase, *STS* stachyose synthase. Abbreviations for the remaining enzymes are listed in Table 2

Through our analysis of the fenugreek EST database and the previous work of others (Naoumkina et al. 2007; Reiter 2008; Seifert 2004), we were able to identify genes likely to be involved in galactomannan biosynthesis and propose a model for the pathway leading to its production (Fig. 3). Table 2 summarizes EST profiling data for these genes, including numbers of EST reads totaled from the four libraries as well as expression levels.

**Table 2:** Putative fenugreek endosperm-expressed genes involved in galactomannan biosynthesis

Putative fenugreek gene	Number of ESTs	*Arabidopsis*	Expression (ppm)			
		Best hit	20 DPA	25 DPA	28 DPA	32 DPA
*SUS* (sucrose synthase)	(4,043)^a^					
*SUS1*	2,408	*At3g43190*	1,668	1,597	1,561	1,650
*SUS2*	1,354	*At3g43190*	1,823	725	621	426
*SUS3*	281	*At4g02280*	307	93	177	166
*FK* (fructokinase)	(270)^a^					
*FK1*	180	*At2g31390*	101	163	96	136
*FK2*	90^b^	*At3g59480*	107	59	38	41
*PMI* (phosphomannose isomerase)	377	*At3g02570*	104	179	334	396
*PMM* (phosphomannomutase)	924	*At2g45790*	294	476	715	1,041
*GMP* (GDP-mannose pyrophosphorylase)	(5,743)^a^					
*GMP1*	3,584^c^	*At2g39770*	770	2,619	2,939	3,345
*GMP2*	2,159	*At2g39770*	259	1,252	1,901	2,439
*UGE* (UDP-glucose/UDP-galactose 4-epimerase)	(1,313)^a^					
*UGE1*	849	*At1g12780*	366	506	676	727
*UGE2*	464	*At4g10960*	189	467	342	233
*ManS* (mannan synthase)	6,230^b^	*At5g03760*	500	2,885	5,031	8,934
*GalT* (galactosyl transferase)	(16,585)^a^					
*GalT1 (GMGT)*	14,239^b^	*At2g22900*	2,761	10,785	11,641	13,285
*GalT2*	2,346	*At2g22900*	358	1,398	2,305	2,121

*GDP* guanosine diphosphate, *UDP* uridine diphosphate

^a^Total number of EST reads added from that of multiple expressed genes

^b^Total number of EST reads added from that of two different contigs corresponding to the same gene

^c^Total number of EST reads added from that of three different contigs corresponding to the same gene

The model predicts that sucrose entering the developing seed is used by sucrose synthase (SUS) to produce fructose (Fru) and UDP-Glc (Fig. 3). The transcript levels of SUS are very high, whereas the transcript levels of invertase (INV) are very low (Table 2; data not shown). Fru appears to be metabolized to Man-1-P which is subsequently converted to GDP-Man, because expression levels at late ages for enzymes acting from Fru to GDP-Man increased (Fig. 3; Table 2).

For three of the enzymes proposed to act in the galactomannan biosynthetic pathway [phosphomannose isomerase (PMI), phosphomannomutase (PMM) and ManS], a single expressed gene was identified. For the remaining five enzymes, two genes were expressed for fructokinase (FK), GDP-Man pyrophosphorylase (GMP), UDP-Glc/UDP-Gal 4- epimerase (UGE) and galactosyl transferase (GalT), and three genes were expressed for sucrose synthase (SUS). With the exception of *FK* and *SUS* genes, expression of which either remained constant or decreased with age, most of the genes were up-regulated during fenugreek seed development, and *GMP*, *GalT2*, *PMM*, *PMI* and *UGE1* showed similar expression patterns as *ManS* and *GMGT* (*GalT1*) (Supplemental Fig. 2b; Table 2).

The EST profiling data for *ManS* and *GalT* were consistent with the quantitative RT-PCR results (Fig. 2b), and the results from Northern blot analysis (Supplemental Fig. 1). Of the two identified endosperm-expressed putative *GalT* genes (named *GalT1* and *GalT2*), *GalT1* was the same gene as the previously identified and characterized *GMGT* (Edwards et al. 1999, 2002) and as used for quantitative RT-PCR analysis (Fig. 2b). Both EST profiling and quantitative RT-PCR analysis showed that the expression levels of *ManS* and *GMGT* increased from 20 to 32 DPA (Fig. 2b; Table 2).

It is known that Gal-containing oligosaccharides are present in trace amounts in fenugreek seeds, including raffinose and stachyose (Campbell and Reid 1982; Reid and Meier 1970b). Raffinose is the galactosyl sucrose trisaccharide, and stachyose is the digalactosyl sucrose tetrasaccharide. Raffinose is synthesized by raffinose synthase using sucrose and galactinol as substrates, and stachyose is synthesized from raffinose at the presence of galactinol by stachyose synthase (Peterbauer and Richter 2001). Raffinose content decreases, whereas a small amount (about 10 μg per endosperm) of stachyose is accumulated alongside large quantities (2–3 mg per seed) of galactomannans in the endosperm during fenugreek seed development (Campbell and Reid 1982; Reid and Meier 1970b). The very small EST read numbers for genes encoding the two enzymes (Fig. 3) are consistent with the low contents of the oligosaccharides in fenugreek endosperms.

Because galactomannans are the predominant polysaccharide accumulated in the endosperm, genes encoding biosynthetic enzymes of other polysaccharides were expected to be expressed at a very low level. Not surprisingly, the expression levels of genes for cellulose synthase, and in particular, xyloglucan biosynthetic enzymes, were much lower than those for ManS and GMGT (Supplemental Table 2; Table 2).

In addition to genes encoding enzymes potentially acting in the galactomannan biosynthetic pathway, we also identified genes encoding one sucrose transporter and a number of putative nucleotide sugar transporters (NSTs) (Supplemental Table 3). Two (named *NST1* and *NST2*) of the putative *NST* genes were highly expressed in the endosperm. NST1 was most similar to the *Arabidopsis* UDP-Gal transporter AtNST-KT (Rollwitz et al. 2006), and NST2 had sequence similarity to both the UDP-Gal transporter AtUDPGalT1 (Bakker et al. 2005) and the putative GDP-Man transporter AtGONST5 (Handford et al. 2004) of *Arabidopsis*. *NST2* was up-regulated, whereas the expression level of *NST1* remained constant or slightly decreased, during fenugreek endosperm development.

Deep EST sequencing of fenugreek endosperms allowed us to identify many transcription factor genes (Supplemental Table 4). Among them were two NAC domain containing protein genes (*NAC10* and *NAC75*). Both genes were relatively highly expressed in the fenugreek seed endosperm. NAC family transcription factors are only present in plants, and some of them have been demonstrated to function as master regulators of secondary wall formation (Zhong et al. 2010). Because galactomannans are deposited in the secondary wall of fenugreek endosperm cells, the two fenugreek NAC transcription factors are promising candidates for involvement in the regulation of galactomannan biosynthesis.

### In vitro enzymatic activity assays of endosperm ManS and GMGT

**Fig. 4:**
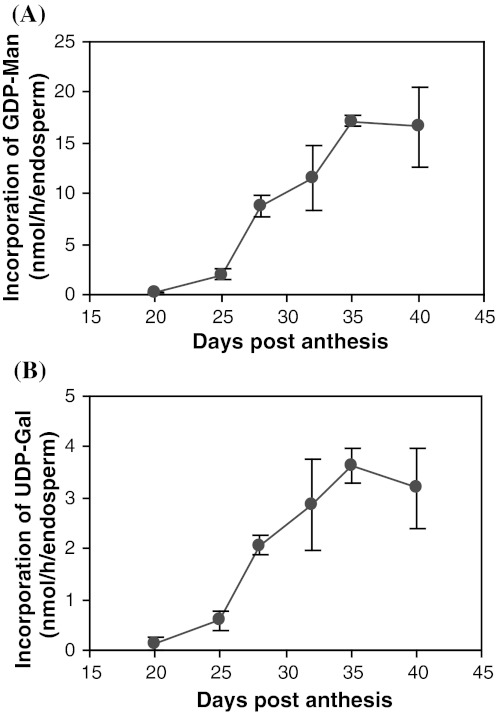
In vitro enzymatic activities of endosperm ManS (**a**) and GMGT (**b**). The activities are shown as nanomoles of GDP-mannose (**a**) and UDP-galactose (**b**) incorporation per hour per endosperm (nmol/h/endosperm). Data for each age are from three biological replicates, 4–14 endosperms from one pod of one individual plant per replicate. *Error bars* represent the standard deviation

Earlier work by Edwards et al. (1992) described in vitro assays to measure the enzymatic activities of endosperm ManS and GMGT during seed development. In order to test whether the enzymatic activities of the encoded proteins correlated with the transcript levels, we utilized these assays to measure ManS and GMGT activities in endosperm homogenates at six ages, including the same four ages (20, 25, 28 and 32 DPA) as used for the EST profiling (Fig. 4). Both ManS and GMGT activities increased slowly from 20 to 25 DPA, but then increased rapidly after 25 DPA, peaked at 35 DPA, and slightly decreased from 35 to 40 DPA. This pattern is similar to that previously reported (Edwards et al. 1992) except that ManS and GMGT activities peaked earlier (35 DPA instead of 40 DPA). The activity data are consistent with the observation that galactomannan deposition also peaked earlier under our growth conditions. Therefore, the levels of ManS and GMGT activities increased as expected.

### In vitro enzymatic activity assays of ManS, GlcS and GlcManS to test substrate specificity

Previous structural analysis (Andrews et al. 1952; Reid and Meier 1970a) has shown that the backbone of fenugreek galactomannans consists of Man and lacks Glc found in many mannan polysaccharides, consistent with our neutral monosaccharide composition data (Fig. 1b). To explore the cause for this observation, we investigated the substrate specificity of the ManS found in fenugreek endosperm. ManS, GlcS and GlcManS assays were performed in vitro using isolated fenugreek endosperm microsomes (Table 3). When the fenugreek microsomes were assayed using GDP-Man as the substrate, incorporation into 70 % ethanol-insoluble products was more than 300 times higher than when GDP-Glc was used as the substrate. In the presence of the two substrates, the incorporation of GDP-Man was approximately 7 times higher than that of GDP-Glc. The presence of GDP-Man promoted incorporation of GDP-Glc, whereas the presence of GDP-Glc suppressed incorporation of GDP-Man. These results suggest that fenugreek endosperm ManS preferentially uses GDP-Man as the substrate for backbone synthesis.

**Table 3:** Mannan synthase (ManS), glucan synthase (GlcS) and glucomannan synthase (GlcManS) activities of microsomes isolated from fenugreek endosperms or *Pichia* cells

Assay	Substrate	Activity (pmol/mg protein/h)				
		Fenugreek endosperm		Microsomes of *Pichia* cells expressing		
		Microsomes	Boiled	TfManS	AtCslA9	AtFUT1
ManS	GDP-Man	6903.5 ± 322.6^a^	0.2 ± 15.9	4274.6 ± 47.9^a^	3034.3 ± 95.4^a^	613.6 ± 6.8
GlcS	GDP-Glc	20.3 ± 26.8	0.1 ± 3.5	23.0 ± 2.6	86.8 ± 5.9^a^	24.0 ± 1.7
Glc*ManS	GDP-Man, GDP-Glc*	186.9 ± 42.1^a^	10.7 ± 7.7	52.5 ± 18.4^a^	152.5 ± 1.5^a^	14.8 ± 2.3
GlcMan*S	GDP-Man*, GDP-Glc	1238.9 ± 28.7^a^	2.3 ± 8.1	733.1 ± 25.3^a^	1408.1 ± 58.5^a^	578.9 ± 7.1

All substrates were at a final concentration of 25 μM. Asterisks indicate the labeled nucleotide sugar substrate used in GlcManS reactions. Values are mean ± SD (n = 2)

^a^Significantly different from the boiled microsomes control or from the AtFUT1 control (*P* < 0.03)

When expressed heterologously in *Pichia*, TfManS showed a much higher degree of substrate preference for GDP-Man than AtCSLA9, an *Arabidopsis* CSLA protein which has been shown to have GlcManS activity in vitro (Liepman et al. 2005). In addition, the presence of GDP-Glc more significantly suppressed incorporation of GDP-Man by TfManS than that by AtCSLA9 (Table 3).

### Analysis of sugar phosphates and nucleotide sugars in endosperms

To further understand biochemical details underlying specific accumulation of a single polysaccharide (galactomannans) in the seed endosperm and evaluate our model for galactomannan biosynthetic pathway (Fig. 3), we analyzed the levels of sugar phosphates and nucleotide sugars in the endosperm of developing fenugreek seeds by using a new method that was recently reported elsewhere (Alonso et al. 2010).

**Fig. 5:**
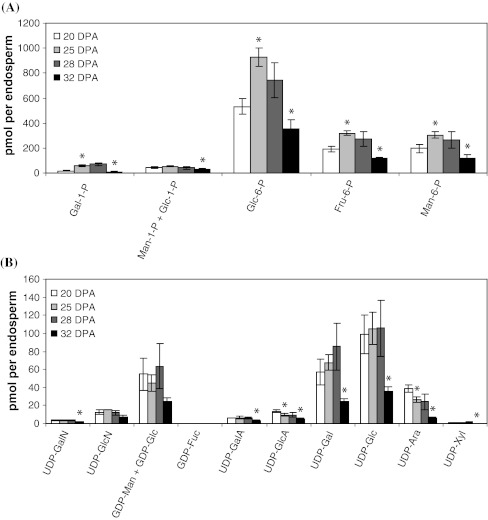
Contents of hexose phosphates and nucleotide sugars per endosperm at four different ages. Fenugreek endosperms were sampled as described in Materials and Methods. Intracellular metabolites were extracted in boiling water, filtered and concentrated before being analyzed by LC–MS/MS using the separation and hardware described previously (Alonso et al. 2010). **a** Hexose phosphate composition; **b** nucleotide sugar composition in fenugreek endosperms. The *bars* for each stage are the mean ± SD from three biological replicates, 7–17 endosperms from one pod of one individual plant per replicate. For each compound, *asterisks* indicate statistically significant differences between two stages (*P* < 0.05). In (**b**), GDP-Man and GDP-Glc could not be separated from each other, and their combined level (GDP-Man + GDP-Glc) was shown. *Fru* fructose, *GalA* galacturonic acid, *GalN* acetylgalactosamine, *GDP* guanosine diphosphate, *GlcA* glucuronic acid, *GlcN* acetylglucosamine, *P* phosphate, *pmol* picomoles, *UDP* uridine diphosphate

Glc-6-P, Fru-6-P and Man-6-P were the three predominant sugar phosphates in the fenugreek endosperm, with maximum levels of 928, 320 and 305 pmol per endosperm respectively at 25 DPA (Fig. 5a). The predominant nucleotide sugars were UDP-Glc, UDP-Gal and the combined GDP-Man and GDP-Glc (GDP-Man + GDP-Glc), which reached maximum levels of 106, 85 and 63 pmol per endosperm respectively at 28 DPA (Fig. 5b). The levels of Gal-1-P, Glc-6-P, Fru-6-P and Man-6-P significantly increased from 20 to 25 DPA. In contrast, the levels of most of the nucleotide sugars remained constant from 20 to 28 DPA (Fig. 5). For both sugar phosphates and nucleotide sugars, contents significantly decreased between 28 and 32 DPA (Fig. 5), corresponding to the period of active galactomannan accumulation (Fig. 1).

## Discussion

Accumulation of cell wall polysaccharides as storage carbohydrates occurs widely in seeds. The storage carbohydrates are massively deposited as a single type of polysaccharide during seed development and are mobilized following seed germination (Reid 1985). These seeds have been used as effective models for studying cell wall polysaccharide biosynthesis due to high-level expression and high activities of the relevant biosynthetic enzymes. One good example is the seeds of many leguminous plants including fenugreek, guar and carob, which accumulate high levels of galactomannans specifically in the endosperm (Reid 1985). Earlier studies using this model system have led to identification, cloning and characterization of *ManS* in guar (Dhugga et al. 2004) and *GMGT* in fenugreek (Edwards et al. 1999).

In this work, we used fenugreek seeds as a model system to further study galactomannan biosynthesis. The specificity of fenugreek endosperms in accumulation of galactomannans as a single polysaccharide was confirmed by several sets of data. First, Man and Gal were the predominant monosaccharides in seed endosperms (Fig. 1b), whereas their contents were negligible in embryos and seed coats (data not shown). Second, *GMGT* and *ManS* were among the 10 most highly expressed genes (Table 1), and were specifically expressed in seed endosperms (Fig. 2a). Third, compared with *ManS* and *GMGT*, genes encoding enzymes for biosyntheses of cellulose, and in particular, xyloglucan, have much lower expression, with the transcript levels of most cellulose synthase genes decreasing during active galactomannan deposition (Table 1; Supplemental Table 2). Fourth, compared with the data from *Arabidopsis* suspension culture cells that mainly produce cellulose, fenugreek endosperms had significantly higher percentages of Gal-1-P, Man-6-P, UDP-Gal and GDP-Man + GDP-Glc and lower percentages of Glc-6-P and UDP-Glc (Compare Fig. 5 with data from Alonso et al. 2010).

Like that of other plant wall polysaccharides, biosynthesis of galactomannans is a complicated process because it is closely connected to central metabolism and involves multiple reactions, which occur in different cellular compartments. GDP-Man and UDP-Gal are synthesized in the cytosol, and then transported by putative nucleotide sugar transporters into the Golgi, where they are used as precursors for galactomannan biosynthesis (Seifert 2004). Therefore, galactomannan biosynthesis needs the coordinated action of many enzymes and transporters in addition to ManS and GMGT, which synthesize the polymer. The same transcription factor networks may coordinately regulate some of the genes encoding these proteins in order to more efficiently synthesize galactomannans. Therefore, understanding of galactomannan biosynthetic pathway and its regulatory mechanism necessitates identification of genes for these related proteins.

Earlier efforts to study galactomannan biosynthesis by EST profiling of guar seeds have been made using the traditional Sanger sequencing technology. Dhugga et al. (2004) first reported sequencing of approximately 15,000 ESTs from guar seeds, and as a result, they identified the *ManS* gene as a member of the *CSLA* gene family. However, the sequence data from this study are not publically available with the exception of the guar *ManS* cDNA sequence. Naoumkina et al. (2007) sequenced approximately 16,500 guar seed ESTs to identify additional genes for galactomannan metabolism. All the EST sequences are available at GenBank. However, because the ESTs were derived from whole seeds instead of seed endosperms it is difficult to determine which transcript increases are correlated with galactomannan biosynthesis. Moreover, because of the limited number of ESTs these datasets do not allow for the identification of lowly expressed genes proposed to act or be involved in galactomannan biosynthesis, including those encoding nucleotide sugar transporters and transcription factors.

To identify more genes involved in galactomannan biosynthesis and its regulation, especially those with low transcript abundance, we used next generation sequencing technology (454 sequencing) to accomplish deep EST profiling of fenugreek endosperms. By this approach, we generated a total of 1.5 million EST reads, analysis of which made it possible to identify genes expressed at a low level. The raw EST short reads data (accession numbers SRX026988–SRX026992) were deposited in the Sequence Read Archive at the National Center for Biotechnology Information (http://www.ncbi.nlm.nih.gov/), and information about consensus sequences of assembled contigs and their annotation is available at the website http://glbrc.bch.msu.edu/fenugreek. These publically available data will provide useful resources for the scientific community to further investigate the details of galactomannan biosynthesis and regulation of this process.

Based on annotation of the consensus sequences, we were able to identify fenugreek genes (Fig. 3; Table 2) whose homologs in *Arabidopsis* or other plant species are known to act in the galactomannan biosynthetic pathway, and to examine their expression levels at the times before, at the beginning of, and during active galactomannan accumulation (Fig. 1; Table 2). The deep EST profiling data were validated by both quantitative RT-PCR analysis of *ManS* and *GMGT* (Fig. 2) and the activity assays of both enzymes (Fig. 4).

Our EST profiling analysis revealed only one gene expressed for PMI, PMM and ManS and at least two genes expressed for the remaining enzymes, including GalT. Most of the genes involved in galactomannan biosynthesis were up-regulated during seed development; the exceptions were *SUS* and *FK* genes, expression of which either remained constant or decreased with age (Supplemental Fig. 2b; Table 2). Cluster analysis (Eisen et al. 1998) revealed that *GalT2*, *GMP*, *PMM*, *PMI* and *UGE1* showed a similar expression pattern as *ManS* and *GMGT* (Supplemental Fig. 2b). It is not known how many genes for each enzyme are present in fenugreek. Because multiple *ManS* genes have been found in all sequenced genomes of land plant species (Yin et al. 2009), we predict that there should be more than one *ManS* gene in fenugreek. Out of the two *GalT* genes, *GalT1* was more highly expressed, and is the same gene as that previously identified and characterized by Edwards et al. (1999; 2002). *GalT2* was expressed at a relatively high level (Table 2). The two GalT isozymes might coordinately act to contribute to a high degree (up to 90 %) of galactosyl substitutions of the mannan backbone. It will be interesting to test whether GalT2 function in adding galactosyl side chains to the mannan backbone, as does GalT1.

In addition to enzymes likely to act in the galactomannan biosynthetic pathway, we also identified other proteins potentially involved in galactomannan metabolism such as transporters and an unknown protein containing DUF246. Three highly expressed putative transporters identified include a sucrose transporter (sucrose-proton symporter) and two nucleotide sugar transporters (NST1 and NST2) (Supplemental Table 3). NST1 was a close homolog of the *Arabidopsis* UDP-Gal transporter AtNST-KT (Rollwitz et al. 2006), and NST2 showed high sequence similarity to both the UDP-Gal transporter AtUDPGalT1 (Bakker et al. 2005) and the putative GDP-Man transporter AtGONST5 (Handford et al. 2004) of *Arabidopsis*. A recent topology study has revealed that AtCSLA9, an *Arabidopsis* ManS, is localized to the Golgi with its active site facing the lumen (Davis et al. 2010), which suggests that GDP-Man and UDP-Gal synthesized in the cytosol need to be transported to the Golgi lumen probably through transporters. Because the nature of nucleotide sugar transporters cannot be accurately predicted based on sequence similarity (Reyes and Orellana 2008), it remains to be determined whether NST1 and NST2 function as a GDP-Man or UDP-Gal transporter. Efforts to evaluate the function of these transporters are underway.

*DUF246* was the second most highly expressed gene identified (Table 1). Preliminary analysis shows that it is specifically expressed in fenugreek seed endosperm, and mutations in its two *Arabidopsis* orthologous genes cause reduction at the mannan level and ManS activity (Yan Wang, Jennifer C. Mortimer, Paul Dupree and Kenneth Keegstra, unpublished results), suggesting that DUF246 is involved in mannan biosynthesis. The related data will be reported in another publication. Some of the *Arabidopsis* DUF246 proteins were predicted to be glycosyltransferases (Hansen et al. 2009). If the fenugreek DUF246 protein is a glycosyltransferase, it might be involved in primer synthesis of mannans or function in the glycosylation of ManS or a ManS-interaction protein to promote the stability, and/or enhance the activity, of ManS.

Mannans are widespread among land plants, and *CSLA* genes have been found in all plants studied (Yin et al. 2009), suggesting that the machinery for mannan biosynthesis is present in all plants. The machinery is generally turned off or operates at a low level because only small amounts of mannans are present in the walls of most angiosperms. However, large quantities of mannans are accumulated as storage carbohydrates in seeds of many plants in different families, indicating that the machinery has been turned on independently many times during plant evolution (Pauly and Keegstra 2010). We speculate that there must be one or more master regulators controlling the regulatory network leading to galactomannan deposition. The finding that most of the genes involved in the substrate (GDP-Man and UDP-Gal) biosyntheses and *GalT2* were up-regulated like *ManS* and *GMGT* (Supplemental Fig. 2b) makes our speculation plausible. Through the high throughput EST sequencing, we discovered a number of transcription factors expressed in fenugreek endosperms (Supplemental Table 4). Interestingly, two NAC family transcription factor genes (*NAC10* and *NAC75*) were expressed at a relatively high level in the endosperm. Because some NAC family transcription factors are implicated in the regulation of secondary cell wall (Zhong et al. 2010), the two fenugreek NAC transcription factors are good candidates for regulation of galactomannan biosynthesis. We are examining whether any of them are involved in regulating galactomannan biosynthesis, either in fenugreek or other systems that synthesize mannans and galactomannans.

Identification of fenugreek genes likely to be involved in galactomannan biosynthesis has allowed us to propose a model for its pathway operating in the fenugreek endosperm (Fig. 3). Our model was built on the previous one proposed by Naoumkina et al. (2007) through EST sequencing of developing guar seeds. In their work, few ESTs (2–11) were identified for most enzymes, and no EST was found for the remaining enzymes. In our model (Fig. 3), galactomannan is synthesized using sucrose as the source of reduced carbon. Sucrose is transported from source leaves via phloem to sink tissues, endosperm in the case of fenugreek seeds. We predict that in the endosperm tissue, sucrose is metabolized by SUS because the transcript level of *SUS* was much higher than that of *INV* (Fig. 3; Table 2; data not shown). These data are consistent with the predominant path of sucrose cleavage by SUS found in sink tissues such as root, tuber, fruit and seed at the storage and maturation stages (Claeyssen and Rivoal 2007; Koch 2004). Total EST reads as well as expression levels at late ages for enzymes acting from Fru to galactomannans or from UDP-Glc to galactomannans increased (Fig. 3; Table 2), suggesting that the carbon flux is strongly directed towards galactomannan production. Genes encoding most of the enzymes involved in substrate (GDP-Man and UDP-Gal) biosyntheses were found to be up-regulated along with *ManS* and *GMGT* (Supplemental Fig. 2b) to achieve efficient galactomannan synthesis.

The model is consistent with our sugar metabolite composition data (Fig. 5). The levels of hexose phosphates significantly increased (20–25 DPA) before, and decreased (25–32 DPA) during, active accumulation of galactomannans (Figs. 1 and 5), which correlates well with dramatic increase of ManS and GalT activities (Fig. 4). However, the levels of nucleotide sugars remained almost constant before they decreased at the middle stage (28–32 DPA) of active galactomannan accumulation (Figs. 1 and 5). These data suggest that fenugreek endosperms mainly used existing pools of hexose phosphates to produce nucleotide sugar precursors necessary for galactomannan synthesis. The amount of sugar phosphates converted to nucleotide sugars was low in the beginning, but increased with active galactomannan accumulation probably due to increased levels of the related enzymes, as reflected by their increased transcript levels (Table 2). At the late stage (32DPA) of active galactomannan accumulation, the limited supply, and thus decreased breakdown, of sucrose might become a limiting factor for the whole pathway. Based on these results, we conclude that active incorporation of GDP-Man and UDP-Gal into galactomannans is the driving force for the whole galactomannan biosynthetic pathway. However, a metabolite flux analysis (Allen et al. 2009; Alonso et al. 2011; Dieuaide-Noubhani et al. 2007; Libourel and Shachar-Hill 2008; Ratcliffe and Shachar-Hill 2006; Schwender 2008; Schwender et al. 2004) in developing fenugreek endosperms is needed to convincingly test our model and identify key steps in the pathway.

Among nucleotide sugars, UDP-Glc had the highest level in the endosperm. This has also been found in *Arabidopsis* suspension culture cells (Alonso et al. 2010). The reason may be that UDP-Glc is a central intermediate in the formation of other nucleotide sugars, including the UDP-Gal used for galactomannan biosynthesis.

Our monosaccharide composition analysis (Fig. 1b) showed that the backbone of fenugreek galactomannans consists of Man and lacks the glucosyl residues found in the backbone of other mannan polysaccharides, consistent with results from other studies (Andrews et al. 1952; Reid and Meier 1970a). In vitro enzymatic assays revealed that fenugreek endosperms preferentially used GDP-Man as the substrate for the backbone synthesis (Table 3). This result was confirmed with fenugreek ManS (TfManS) heterologously expressed in *Pichia*. Compared with AtCSLA9 which has been shown to have GlcManS activity in vitro (Liepman et al. 2005), TfManS showed a much higher degree of substrate preference for GDP-Man, and incorporation of GDP-Man by TfManS was more strongly suppressed by the presence of GDP-Glc (Table 3).

Because fenugreek endosperm microsomes could weakly incorporate GDP-Glc in the presence of GDP-Man in vitro (Table 3), one possible cause for the lack of Glc in the backbone of fenugreek galactomannans could be the absence of GDP-Glc in fenugreek endosperms, in addition to the strong substrate preference of fenugreek ManS for GDP-Man. Absence of GDP-Glc may be evolutionally beneficial to efficient biosynthesis of galactomannans because it prevents the inhibitive effect of GDP-Glc on the incorporation of GDP-Man by ManS (Table 3). However, we cannot test this notion, because GDP-Glc could not be separated from GDP-Man in our analysis of nucleotide sugars (Fig. 5).

In summary, through deep EST sequencing, we have identified a cohort of genes likely to be involved in galactomannan biosynthesis, in addition to the known *ManS* and *GMGT* genes, and proposed a model for the biosynthetic pathway, which is consistent with our sugar metabolite composition data. Based on our results, we concluded that the fenugreek endosperm tissue has evolved the special function of exclusively producing galactomannans probably by up-regulation of genes encoding most enzymes involved in the galactomannan biosynthesis and other related proteins, and down-regulation or suppression of most, if not all, genes for biosyntheses of other cell wall polysaccharides. In addition, fenugreek endosperms preferentially used GDP-Man as the substrate to synthesize mannans as the backbone instead of glucomannans in vitro. We are investigating the function of the newly identified genes including *DUF246* and putative *NSTs*, and dissecting the transcriptional regulatory network controlling galactomannan biosynthesis. The latter is a complex process that will take many years of investigation to untangle the many regulatory networks that control the deposition of specific cell wall polysaccharides.

Galactomannans have important practical applications and are a potential candidate for improving plant wall feedstock for biofuel production (Pauly and Keegstra 2008; Srivastava and Kapoor 2005). Increasing galactomannan levels in crop plants cannot be accomplished by simply manipulating the expression level of the backbone synthetic gene (Naoumkina et al. 2008), and thus needs a complete understanding of galactomannan biosynthetic pathway and its regulation. Our work provides information and resources toward this goal.

## Electronic supplementary material

Below is the link to the electronic supplementary material.

Supplementary material 1 (PDF 282 kb)

Supplementary material 2 (XLS 766 kb)
