# Supplementary material for: Deep EST profiling of developing fenugreek endosperm to investigate galactomannan biosynthesis and its regulation
Source: Plant Mol Biol. 2012 Apr 17;79(3):243–58. doi: 10.1007/s11103-012-9909-y (PMC3349874; doi:10.1007/s11103-012-9909-y)
Supplement: Supplementary file 1 — Supplementary material 1 (PDF 282 kb) [file 11103_2012_9909_MOESM1_ESM.pdf]

Electronic supplementary material

Deep EST profiling of developing fenugreek endosperm to investigate galactomannan biosynthesis and its regulation

Plant Molecular Biology

Yan Wang, Ana P. Alonso, Curtis G. Wilkerson, Kenneth Keegstra

Corresponding Author:

Kenneth Keegstra
MSU-DOE Plant Research Laboratory, Michigan State University, East Lansing, MI 48824, USA
E-mail: keegstra@msu.edu

Supplementary material 1

Supplemental methods

Supplemental Fig. 1 Northern blot analysis of *ManS* and *GMGT*

Supplemental Fig. 2 Cluster analysis of transcript levels of fenugreek endosperm-expressed genes at different ages

Supplemental Table 2 Fenugreek endosperm-expressed genes encoding putative enzymes for biosyntheses of cellulose and xyloglucan

Supplemental Table 3 Fenugreek endosperm-expressed genes encoding putative sucrose and nucleotide sugar transporters

Supplemental Table 4 Fenugreek endosperm-expressed transcription factor genes with at least 100 EST reads

Supplemental references

Supplementary material 2 (as a separate Microsoft Excel document)

Supplemental Table 1 List of fenugreek contigs with at least 50 EST reads

Supplemental methods

Analysis of fenugreek endosperm EST database

The consensus sequences of fenugreek EST contigs and their annotation are available at the website http://glbrc.bch.msu.edu/fenugreek.

To generate the list of contigs with at least 50 EST reads (Supplemental Table 1), the “Minimum ESTs in library” was set to 10, the “Reduced into groups” selection was unchecked, and all of the four libraries were selected. As a result, a list of contigs with 10 or more EST reads found in at least one of the four libraries was shown. The contig list was saved as a Microsoft Excel document (in cvs format) by clicking on the “[Download Results]” link at the upper right corner of the web page. The contigs with less than 50 EST reads (totaled from the four libraries) were then eliminated. For contigs annotated as “No Definition Found”, their consensus sequences were used as queries to search the non-redundant nucleotide sequence database with the BLASTN program (http://blast.ncbi.nlm.nih.gov/Blast.cgi) to identify contigs for rRNA genes. The contigs for rRNA genes were eliminated from the contig list, and the EST abundance (expression level) for the remaining contigs was recalculated and shown as parts per million (ppm). The consensus sequences of the remaining contigs were searched against the non-redundant protein sequence database with the BLASTX program to identify the chimeric contigs (“hybrid” contigs containing sequences from two different genes) due to assembling errors, and to find possible close homologs from other species for contigs with no *Arabidopsis* hit found. The chimeric contigs were then eliminated, and some of the remaining contigs were annotated based on sequence similarity search results.

Expression pattern analysis by Cluster and TreeView

After elimination of contigs for rRNA sequences, we recalculated the expression level of each endosperm-expressed gene, and normalized it as ppm. The expression level of each gene relative to 20 DPA (days post anthesis) was calculated and the resulting ratios were transformed to $\log_2$ values. Gene expression patterns were determined using a hierarchical clustering algorithm as implemented in Cluster (Eisen et al. 1998), with uncentered correlation similarity and average linkage clustering. The results from the Cluster analysis were then visualized as a heat map using TreeView (Eisen et al. 1998).

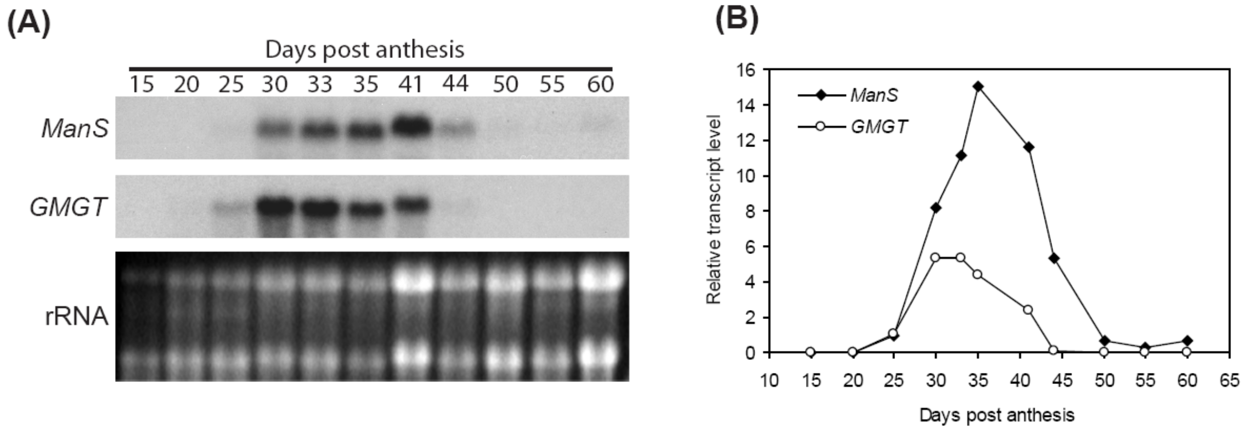

Supplemental Fig. 1 Northern blot analysis of *ManS* and *GMGT*. **a** Northern blot data generated using total RNA from fenugreek developing seeds. **b** Quantification of the data. Relative transcript levels are expressed relative to the 25 DPA (days post anthesis) age

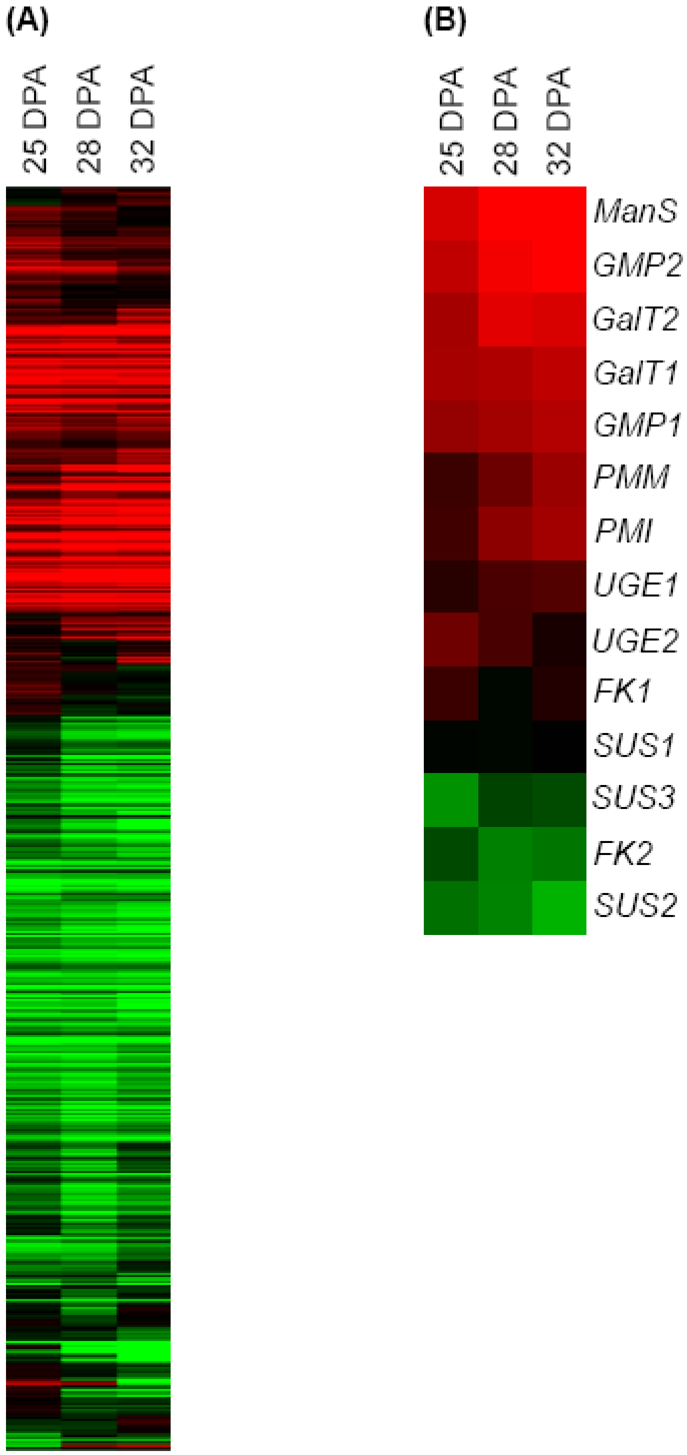

Supplemental Fig. 2 Cluster analysis of transcript levels of fenugreek endosperm-expressed genes at different ages. All measurements are relative to 20 DPA. The color scale is set from saturated green for log_2 ratios -3 and below to saturated red for log_2 ratios of 3 and above. Each gene is shown by a single row of colored boxes, and each age is shown by a single column. Green boxes and red boxes indicate down regulation and up regulation of gene expression during fenugreek endosperm development. **a** Heat map of approximately 1,650 contigs assembled from at least 100 EST reads. **b** Heat map of putative fenugreek genes involved in galactomannan biosynthesis. The designations of the genes are listed in Table 2.

Supplemental Table 2 Fenugreek endosperm-expressed genes encoding putative enzymes for biosyntheses of cellulose and xyloglucan

Rank	Number of ESTs	Putative fenugreek gene	*Arabidopsis* best hit	Expression (ppm)			
				20 DPA	25 DPA	28 DPA	32 DPA
1030	172	Cellulose synthase 2 (*CESA2*)	*At4g39350*	235	82	65	85
1135	156	Cellulose synthase 9 (*CESA9*)	*At2g21770*	251	96	49	16
1808	97	Cellulose synthase 3 (*CESA3*)	*At5g05170*	99	33	48	88
8459	15	Cellulose synthase 1 (*CESA1*)	*At4g32410*	10	14	6	10
8459	15	Cellulose synthase 7 (*CESA7*)	*At5g17420*	40	0	0	0
11443	10	Xyluglucan glucan synthase (*CSLC4*)	*At3g28180*	27	0	0	0
1680	104	Xyloglucan xylosyltransferase 2 (*XT2*)	*At4g02500*	75	59	75	67
13567	8	Xyloglucan galactosyltransferase (*MUR3*)	*At2g20370*	5	3	10	0
8459	15	Xyloglucan fucosyltransferase (*FUT1*)	*At2g03220*	3	14	8	16

If more than one fenugreek contig had the highest similarity to the same *Arabidopsis* gene, only the one with more or the most EST reads is shown.

Supplemental Table 3 Fenugreek endosperm-expressed genes encoding putative sucrose and nucleotide sugar transporters

		Arabidopsis	Expression (ppm)			
Annotation	ESTs	best hit	20 DPA	25 DPA	28 DPA	32 DPA
Sucrose-proton symporter (SUC)	602	At1g71890	375	499	331	447
Nucleotide sugar transporter 2 (NST2)	563	At1g77610	176	347	459	534
Nucleotide sugar transporter 1 (NST1)	501	At4g39390	366	347	321	308
Phosphate translocator-related (pPThKT)	119	At5g25400	32	73	100	115
Transporter-related (pPThKT)	109	At1g34020	80	87	55	77
Transporter-related (pPThKT)	106	At5g42420	113	50	55	71
Phosphate translocator-related (pPThKT)	101	At5g25400	51	100	77	37
Phosphate translocator-related (pPThKT)	81	At5g25400	93	36	35	57
Nucleotide-sugar transmembrane transporter	59	At2g43240	58	40	31	27
Transporter-related (pPThKT)	50	At1g06890	56	30	18	34
Transporter-related (pPThKT)	46	At1g34020	58	33	14	20
Integral membrane family protein (GONST4)	45	At5g19980	30	37	25	30
Triose-phosphate transmembrane transporter (PPT1)	44	At5g33320	61	23	21	10
Organic anion transmembrane transporter (pPThKVAG)	43	At3g11320	67	17	10	24
UDP-galactose transporter 3 (UTR3)	34	At1g14360	13	9	29	41
Nucleotide-sugar transporter family protein	29	At5g41760	48	6	10	14
Nucleotide-sugar transporter family protein	26	At5g65000	35	6	10	20
UTR3 (UDP-GALACTOSE TRANSPORTER 3)	25	At1g14360	0	23	10	41
Glucose-6-phosphate/phosphate translocator 2 (GPT2)	23	At1g61800	16	9	18	16
Phosphate translocator-related (pPThKD)	18	At4g32390	30	17	3	0
Integral membrane family protein (GONST4)	16	At5g19980	8	17	4	16
Glucose-6-phosphate/phosphate translocator 2 (GPT2)	16	At1g61800	5	23	10	4
Phosphate translocator-related (pPThKD)	16	At4g32390	38	3	3	0
Glucose-6-phosphate transporter 1 (GPT1)	15	At5g54800	16	12	4	10
UDP-galactose transporter 6 (UTR6)	13	At3g59360	21	9	4	0
Glucose-6-phosphate/phosphate translocator (UDPGalT1)	12	At1g77610	5	6	6	16

Putative nucleotide sugar transporter (NST) genes were identified by searching the fenugreek EST database using the gene identity (AGI) numbers of known and predicted *Arabidopsis* NSTs summarized by Reyes and Orellana (2008). Putative transporter genes with at least 10 EST reads are shown.

Supplemental Table 4 Fenugreek endosperm-expressed transcription factor genes with at least 100 EST reads

Annotation	ESTs	*Arabidopsis* best hit	Expression (ppm) 20 DPA	25 DPA	28 DPA	32 DPA
HAP5C (heme activated protein 5C); CCAAT family	2221	At1g08970	468	2622	1607	1275
IDD5 (indeterminate(ID)-domain 5); C2H2 family	808	At2g02070	292	715	567	615
L1L (leafy cotyledon 1-related); CCAAT family	685	At5g47670	375	593	469	396
RAP2.3 (related to AP2.3); AP2-EREBP family	537	At3g16770	441	649	273	65
AGL104 (agamous-like 104); MADS family	491	At1g22130	131	376	413	389
HMGB2 (high mobility group B 2); HMG family	443	At1g20693	244	320	286	355
WOX11 (wuschel related homeobox 11); HB family	393	At3g03660	409	283	234	105
HMGB2 (high mobility group B 2); HMG family	370	At1g20693	93	190	382	294
WRKY21 (WRKY DNA-binding protein 21); WRKY family	340	At2g30590	278	236	198	203
NAC10 (NAC domain containing protein 10); NAC family	326	At1g28470	73	202	318	260
ERF/AP2 transcription factor family protein; AP2-EREBP family	290	At1g75490	27	126	248	400
ADA2B/PRZ1 (PROPORZ1); MYB-related family	289	At4g16420	380	227	96	77
HD2C (histone deacetylase 2C); C2H2 family	280	At5g03740	438	123	82	118
Zinc finger (CCCH-type) family protein	278	At5g44260	78	202	223	247
Trihelix DNA-binding protein; Trihelix family	269	At1g76880	152	199	156	233
DNA-binding storekeeper protein-related; GeBP family	264	At1g61730	214	226	118	169
ERF9 (ERF domain protein 9); AP2-EREBP family	262	At5g44210	38	193	244	219
MBF1B (multiprotein bridging factor 1B); MBF1 family	247	At3g58680	80	196	172	230
SHL1 (short life 1); PHD family	240	At4g39100	61	126	213	244
IDD5 (indeterminate(ID)-domain 5); C2H2 family	213	At2g02070	310	163	61	41
LBD41 (LOB domain-containing protein 41); LOB family	206	At3g02550	211	289	44	24
Zinc finger (C2H2 type) family protein	178	At3g02790	21	180	131	152
WOX9 (wuschel-related homeobox 9); HB family	177	At2g33880	222	134	49	81
GeBP family protein	175	At4g25210	131	114	90	148
TLP3 (TUBBY like protein 3); TUB family	173	At2g47900	235	137	55	41
SCL5 (scarecrow-like 5); GRAS family	170	At1g50600	83	76	159	126
bZIP family transcription factor	163	At5g06839	198	93	108	16
SUVH4 (SU(VAR)3-9 homolog 4); SET (PcG) family	162	At5g13960	192	131	62	51
DR1 (TATA-binding protein-associated phosphoprotein Dr1 protein); CCAAT family	153	At5g23090	73	129	113	95
PKL (PICKLE); PHD family	152	At2g25170	275	53	42	37
AP2 domain-containing TF family protein; AP2-EREBP family	149	At4g13040	43	73	121	169
Zinc finger (CCCH-type) family protein; C3H family	149	At2g20280	141	110	77	71
MYB family transcription factor	141	At5g41020	70	107	104	95
AGL66 (agamous-like 66); MADS family	140	At1g77980	3	117	125	132
SUVH1 (SU(VAR)3-9 homolog 1); SET (PcG) family	139	At5g04940	232	56	42	44
ANL2 (anthocyaninless 2); HB family	138	At4g00730	187	79	45	65
HMGB6 (high mobility group B 6); HMG family	138	At5g23420	67	106	92	112
JAZ12/TIFY3B (jasmonate-ZIM-domain protein 12); ZIM family	137	At5g20900	61	84	110	108
LBD41 (LOB domain-containing protein 41); LOB amily	137	At3g02550	30	103	127	101
ARF10 (auxin response factor 10); ARF family	135	At2g28350	121	90	71	85
OBP3 (OBF-binding protein 3); C2C2-Dof family	135	At3g55370	121	90	83	65
AIL7 (aintegumenta-like 7); AP2-EREBP family	134	At5g65510	174	96	39	57
DNA-binding family protein; MYB-realted family	133	At1g72740	163	67	52	81
ARF2 (auxin response factor 2); ARF family	132	At5g62000	123	37	65	142
Zinc finger (CCCH-type) family protein	129	At3g48440	259	37	29	16
DNA binding / transcription factor; GARP-G2-like family	128	At2g38300	113	90	82	55

Supplemental Table 4 (continued)

Putative fenugreek transcription factor	ESTs	*Arabidopsis* best hit	Exression (ppm) 20 DPA	25 DPA	28 DPA	32 DPA
Zinc finger (C2H2 type) family protein; C2H2 family	124	At3g10470	251	70	13	1
Zinc finger (C2H2 type) family protein	120	At3g02790	70	79	69	115
HB23 (homeobox protein 23); ZF-HD family	119	At5g39760	126	47	56	98
STO (salt tolerance); Orphans family	119	At1g06040	91	117	52	67
NAC75 (NAC domain containing protein 75); NAC family	117	At4g29230	106	87	52	75
RAP2.2; AP2-EREBP family	116	At3g14230	86	64	71	95
bZIP transcription factor (bZIP69); bZIP family	115	At1g06070	113	64	77	47
ARF2 (auxin response factor 2); ARF family	110	At5g62000	192	44	25	37
WRKY11 (WRKY DNA-binding protein 11); WRKY family	110	At4g31550	106	67	73	41
PHD finger family protein; Alfin-like family	110	At5g26210	136	59	65	27
HMGB2 (high mobility group B 2); HMG family	110	At1g20693	53	50	62	146
Trihelix DNA-binding protein; Trihelix family	108	At1g76880	139	40	52	57
MBF1A (multiprotein bridging factor 1A); MYB1 family	107	At2g42680	78	73	62	77
HAP2A; CCAAT family	103	At5g12840	86	107	38	55
RWP-RK domain-containing protein; RWP-RK family	102	At4g35590	134	56	49	30
HMGB1 (high mobility group B 1); HMG family	102	At3g51880	43	82	69	85
AGL15 (agamous-like 15); MADS family	100	At5g13790	101	107	29	37

Putative transcription factor (TF) genes were identified by searching the fenugreek EST database using the key word "transcription factor". The list of the TF genes was downloaded from the database website and saved as described in the Supplemental Methods. To confirm the annotations of the TF genes, the identity (AGI) numbers of the *Arabidopsis* best hits were used to search two TF databases (http://plntfdb.bio.uni-potsdam.de/v3.0/ and http://datf.cbi.pku.edu.cn/index.php). If no AGI was found in the databases, the corresponding fenugreek gene was eliminated from the list. Putative TF genes with at least 100 EST reads are shown.

Supplemental references

Eisen MB, Spellman PT, Brown PO, Botstein D (1998) Cluster analysis and display of genome-wide expression patterns. Proc Natl Acad Sci U S A 95:14863-14868

Reyes F, Orellana A (2008) Golgi transporters: opening the gate to cell wall polysaccharide biosynthesis. Curr Opin Plant Biol 11:244-251
